# Direct comparison of retinal structure and function in retinitis pigmentosa by co-registering microperimetry and optical coherence tomography

**DOI:** 10.1371/journal.pone.0226097

**Published:** 2019-12-12

**Authors:** Jun Funatsu, Yusuke Murakami, Shunji Nakatake, Masato Akiyama, Kohta Fujiwara, Shotaro Shimokawa, Takashi Tachibana, Toshio Hisatomi, Yoshito Koyanagi, Yukihide Momozawa, Koh-Hei Sonoda, Yasuhiro Ikeda

**Affiliations:** 1 Department of Ophthalmology, Graduate School of Medical Science, Kyushu University, Fukuoka, Japan; 2 Department of Ophthalmology, Faculty of Medicine, Fukuoka University, Fukuoka, Japan; 3 Laboratory for Genotyping Development, RIKEN Center for Integrative Medical Sciences, Kanagawa, Japan; 4 Department of Ophthalmology, Faculty of Medicine, University of Miyazaki, Miyazaki, Japan; Massachusetts Eye & Ear Infirmary, Harvard Medical School, UNITED STATES

## Abstract

**Purpose:**

To evaluate the retinal structure-function relationships in the macula of retinitis pigmentosa (RP) patients by comparing microperimetry-3 (MP-3) images with co-registered optical coherence tomography (OCT) images.

**Methods:**

Thirty patients with typical RP were recruited from our hospital. The maculae of patients were examined with MP-3 and OCT. The retinal sensitivity was measured by MP-3 at 40 testing points arranged concentrically in a 16° diameter of the central retina, and we divided the 40 points into four zones according to degree from the fovea (2°, 4°, 6°, and 8°). We analyzed the correlation coefficients between the retinal sensitivity and the total retinal thickness (TRT), the length from the inner limiting membrane to the retinal pigment epithelium (RPE), and between the retinal sensitivity and the outer retinal thickness (ORT), the length from the outer plexiform layer to the RPE at each stimulus point.

**Results:**

TRT showed moderate correlations with the retinal sensitivity at 2° (median ρ = 0.59 interquartile range (IQR) [0.38–0.72]), 4° (ρ = 0.59 [0.55–0.68]) and 6° (ρ = 0.60 [0.54–0.63]), and TRT was weakly-to-moderately related to the retinal sensitivity at 8° (ρ = 0.27 [0.19–0.48]). ORT exhibited strong correlations at 2° (ρ = 0.72 [0.60–0.81]), 4° (ρ = 0.71 [0.75–0.67]) and 6° (ρ = 0.70 [0.54–0.74]), and a weak-to-moderate correlations at 8° (ρ = 0.34 [0.29–0.53]). ORT was more strongly correlated with the retinal sensitivity compared to TRT (p = 0.018).

**Conclusion:**

ORT, rather than TRT, within 6° eccentricity was strongly correlated with the retinal sensitivity, suggesting that measuring ORT in those areas will help evaluate the macular status and progression in RP.

## Introduction

Retinitis pigmentosa (RP) is a group of inherited retinal degeneration disorders characterized by the symptoms of night blindness, visual field (VF) constriction, and a subsequent decline of visual acuity in the later stage [1, 2]. Since a loss of central vision substantially attenuates the quality of life among individuals with RP, a precise evaluation of macular function is important to explain the disease status and progression to patients as well as to evaluate the efficacy of potential therapeutics in clinical trials.

Macular sensitivity in retinal diseases is usually evaluated using static perimetry tests within the central 10° and more recently with the use of microperimetry devices such as the Microperimetry-3 (MP-3; Nidek Technologies, Albignasego, Italy) and the Macular Integrity Assessment (MAIA; CenterVue, Padua, Italy) [3–9]. These microperimetry devices have wide dynamic ranges comparable to that of a Humphrey Field Analyzer (HFA; Carl Zeiss Meditec, Dublin, CA).

A major advantage of microperimetry over conventional perimetry is that microperimetry directly projects light stimuli onto the retina while simultaneously imaging the fundus with auto-tracking, which facilitates the precise measurement of sensitivity at specific retinal points. With the use of an HFA, which does not have an auto-tracking system, it was reported that even healthy subjects are not able to avoid small eye movements [10, 11]. In contrast, MP-3 can accurately measure the retinal sensitivity at the correct position [6]. Asahina et al. reported that compared to the use of HFA 10–2 tests, the retinal sensitivity measured by MP-3 better correlates with ellipsoid zone (EZ) abnormalities in optical coherence tomography (OCT) [12]. Indeed, microperimetry devices have been used as primary or secondary outcome measures in clinical trials for RP (https://clinicaltrials.gov).

Spectral-domain OCT (SD-OCT) offers fine and high-resolution retinal images and is useful for detecting the structural changes in RP such as abnormalities or the disappearance of EZ and thinning of the outer retina, and time-course changes of these parameters [13–20]. Structural indices such as the length or area of the EZ and the thickness of the outer nuclear layer (ONL) or outer segments (OS) are related with visual function in RP. Sayo et al. reported that the ONL/OS thickness is strongly correlated with the retinal sensitivity measured by HFA10-2 tests [21]. However, one limitation is that precise function-structure relationships have not been fully addressed, because the alignment of the retinal sensitivity data with OCT images is difficult when using visual function (VF) data without fundus imaging such as when HFA is used.

For example, Sayo et al. compared the ONL/OS thickness at specific distances from the fovea in horizontal OCT scans with the averaged retinal sensitivity at two points close to (but not at) the target site [21]. In the present study, using a new software program that co-registers OCT en face structural data with the results of microperimetry, we evaluated the direct structure-function relationship in the macula of RP patients.

## Patients and methods

### Ethics statement

This study was approved by the Institutional Review Board of Kyushu University Hospital (Fukuoka, Japan) and was performed according to the tenets of the Declaration of Helsinki on Biomedical Research Involving Human Subjects. Written informed consent was obtained from all participating patients after a thorough explanation of the nature of the study and its possible consequences.

### Patients

We analyzed the cases of 72 patients with RP diagnosed based on the results of a clinical examination, VF measurements, and electroretinography who also underwent MP-3 and OCT imaging. All patients were consecutively recruited at Kyushu University Hospital in 2016 and 2017. The examination results of the right eye of each patient were used for the analyses.

The phenotype of the patients was typical rod-cone dystrophy such as night blindness, progressive concentric VF constriction, and mid-peripheral intra-retinal perivascular “bone-spicule” pigmentary changes and RPE atrophy correlated with arteriolar narrowing. We excluded patients who had complications such as cystoid macular edema, epiretinal membrane with macular traction, and severe cataract and/or whose conditions were associated with other ocular diseases such as glaucoma and uveitis and the comorbid/past retinal vascular diseases. We also excluded the cases of patients with non-reliable MP-3 examination results (false-positive rate <33% and false-negative rate <33%) or low-quality OCT images (e.g., Signal Strength Index scores <6/10), and those for whom the identification of the outer plexiform layer (OPL) was difficult. A final total of 30 RP patients were analyzed. We previously performed the genetic analysis of 83 known RP causative genes for 29 of 30 RP patients [22]. The genetic inheritance patterns were determined based on the detected variants.

### Clinical examination

The best-corrected visual acuity (VA) was measured using a Landolt decimal VA chart (CV-6000: Tomey, Nagoya, Japan; or AVC-36: Kowa, Nagoya, Japan) at 5 m or using single Landolt test cards (HP-1258; Handaya, Tokyo). The values obtained were converted to the logarithm of the minimum angle of resolution (logMAR) units. Fundus autofluorescence (FAF) images were acquired using a Spectralis HRA-OCT (Heidelberg Engineering, Heidelberg, Germany). Automated static perimetry tests were performed by a Humphrey Field Analyzer (HFA) (Carl Zeiss) using the central 10–2 Swedish Interactive Thresholding Algorithm (SITA) Standard Program. The lens was corrected according to the test distance.

### Optical coherence tomography measurement

We used the RS-3000 Advance system (software ver. 2.03, Nidek, Aichi, Japan) to perform the 3D SD-OCT imaging. This instrument includes a confocal scanning laser ophthalmoscope to monitor fundus images and SD-OCT equipment to obtain tomographic images. The OCT equipment has a 7-μm depth resolution in tissue and 20-μm transverse resolution. Each A-scan had a depth of 2.1 mm and comprised 512 pixels, providing a digital depth sampling of 4.1 μm per pixel. For wide-area 3D imaging in the posterior pole, raster scanning over a 6×6-mm square area centered on the foveal center was conducted with a scan density of 512 A-scans (horizontal) × 128 B-scans (vertical). All OCT images consisted of retinal thickness and tomographic mapping.

**Fig 1:**
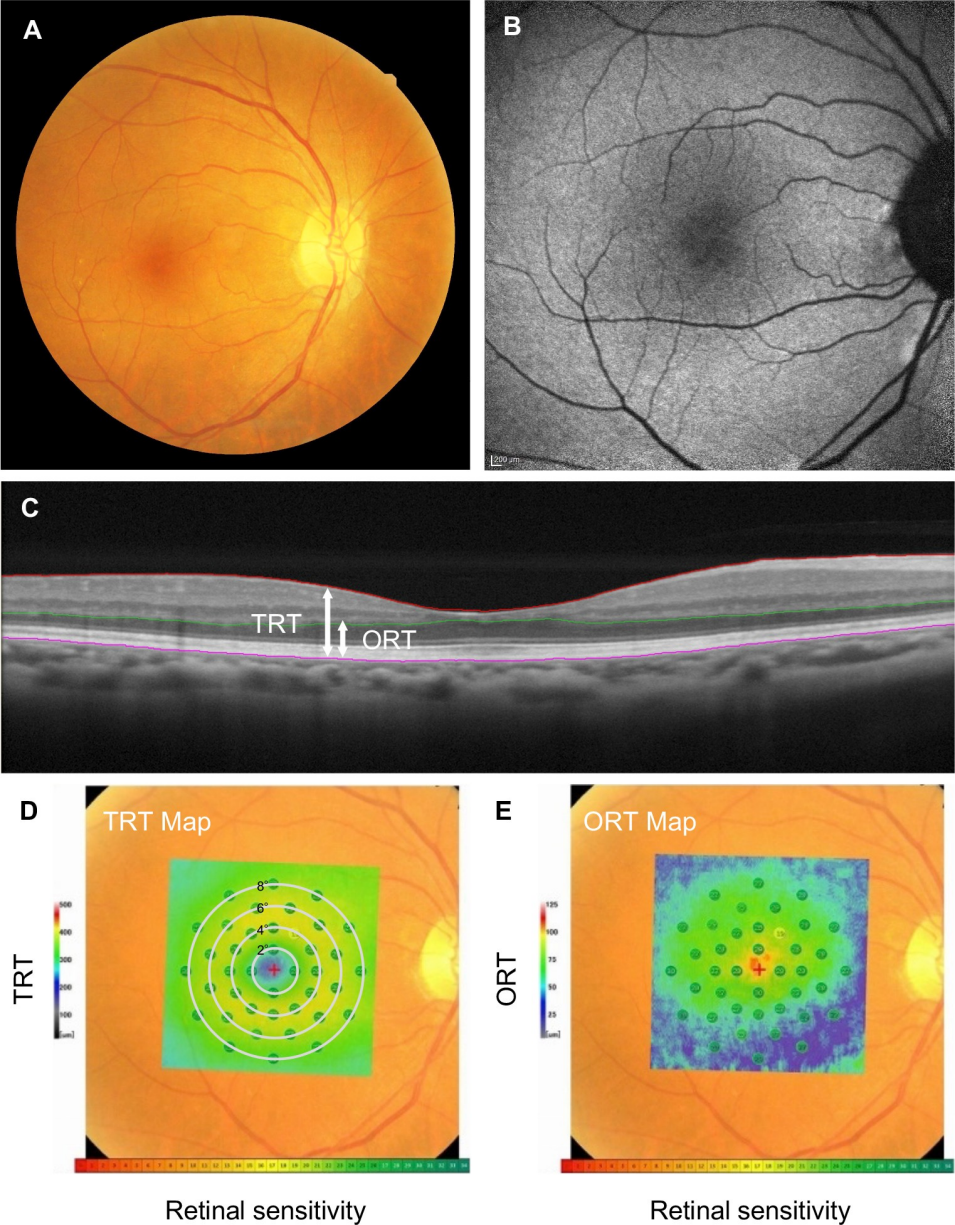
Representative right eye images of a typical RP patient, visual acuity 20/13. **A:** Fundus photo. **B:** FAF. **C:** OCT; “TRT” refers to the thickness between the ILM and the RPE. “ORT” refers to the thickness from OPL to RPE. **D-E.** Co-registered images of fundus photo, microperimetry results, and the ORT/TRT thickness map. The color of the point indicates the retinal sensitivity, and the color of the background indicates the retinal thickness. Regarding the retinal sensitivity, *green* indicates high and *red* indicates low retinal sensitivity. Regarding the retinal thickness, *green* indicates thick and *blue* indicates thin. (**D**: TRT, **E**: ORT).

**Fig 2:**
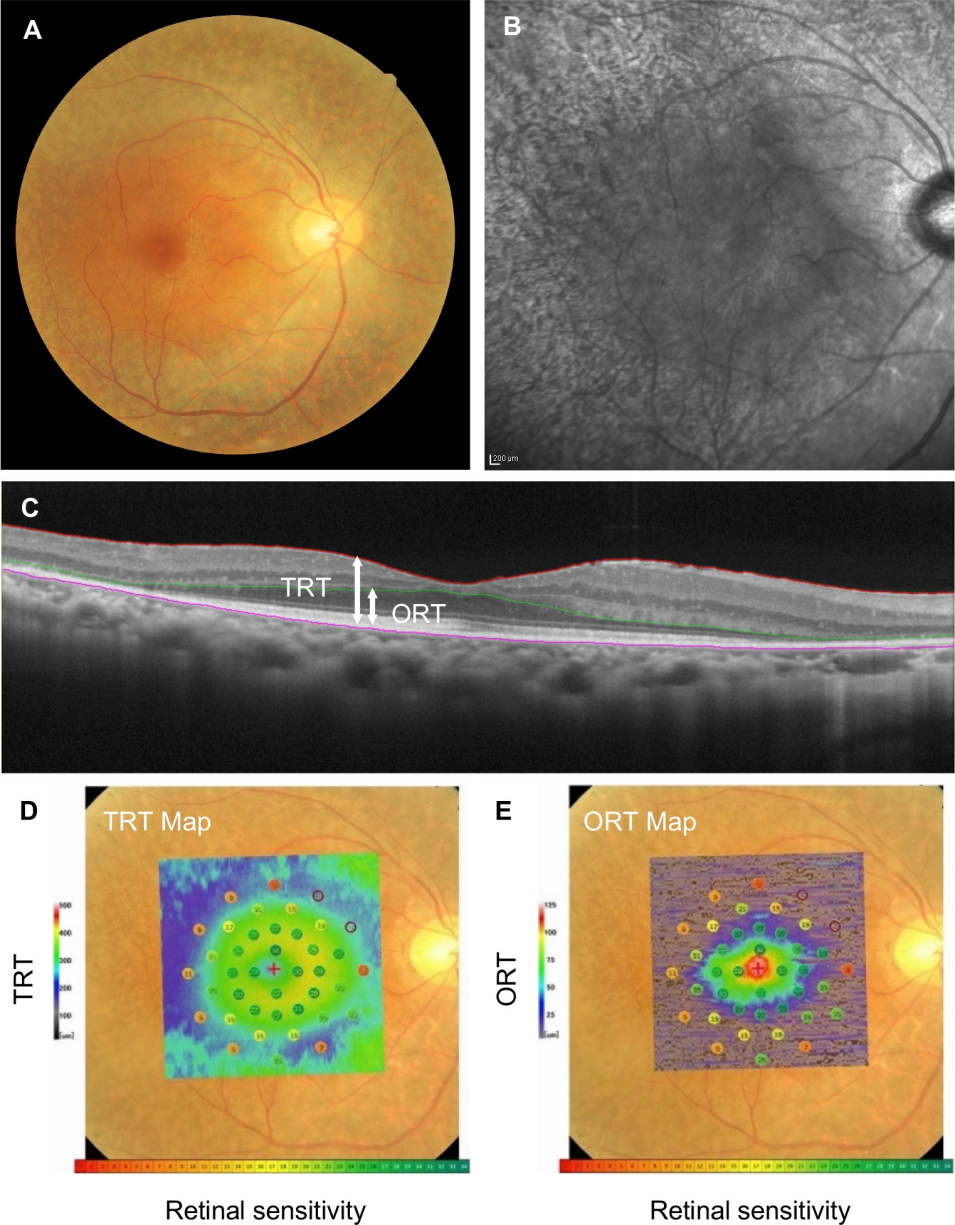
Representative right eye images of a typical RP patient, visual acuity 20/16. **A:** Fundus photo. **B:** FAF. **C:** OCT. **D-E.** Co-registered images of fundus photo, microperimetry results, and the ORT/TRT thickness map. (**D**: TRT, **E**: ORT).

We measured each patient's retinal thickness by using Image Filing Software NAVIS EX (ver.1.5.5) (Nidek). The total retinal thickness (TRT) was defined as the length from the inner limiting membrane (ILM) to the retinal pigment epithelium (RPE). The outer retinal thickness (ORT) was defined as the length from the OPL to the RPE. The layers of ILM, RPE, and OPL were automatically segmented by the software, and thereafter all images were reviewed and manually corrected if incorrect segmentation was observed by a masked observer (S. Nakatake) (Figs 1 and 2).

OCT was performed by a well-trained examiner after pupillary dilatation, with the examiner rejecting any scans with motion artifacts (discontinuous jump), poor centration, poor focus, or missing data and accepting only good-quality images, defined as those with ''Signal Strength Index'' scores (as assigned by the RS3000) of at least 6/10 based on the manufacturer's recommendation.

### MP-3 measurement

The retinal sensitivity of each patient was examined with the MP-3 (Nidek). The MP-3 measurement is similar to HFA measurement on the basis of a 4–2 full threshold staircase strategy. The stimulus dynamic range for the MP-3 was 34 dB. The background luminance is 31.4 asb, and the maximum luminance is 10,000 asb. The MP-3 measurement was carried out using the standard Goldmann III stimulus size and 40 measured testing points within a diameter of 16° of the central retina (Fig 1D and 1E, Fig 2D and 2E). Only reliable VFs were used in the analyses: false-positive rate <33% and false-negative rate <33%. Fixation loss was not applicable, because the MP-3 has an auto-tracking system that enables the projection of the stimulus at predefined retinal positions.

### Evaluation of retinal structure-function relationships using multimodal imaging

Each patient's OCT retinal thickness map was superimposed on the microperimetry data by using Image Filing Software NAVIS EX (Ver.1.5.5) (Nidek). The fundus images obtained with MP-3 and OCT were overlaid based on the position of blood vessel bifurcations as markers. The retinal thickness values (TRT/ORT) at the 40 stimulus points in the MP-3 examination were automatically obtained from the TRT/ORT thickness map, and the data were output as a CSV file. The relationships between the retinal sensitivity and the TRT/ORT at each stimulus point was analyzed. For a subgroup analysis, we divided the 40 points into four zones according to the degree from the fovea (2°, 4°, 6° and 8°; Figs 1 and 2).

### Statistical analyses

The correlation coefficients between the retinal thickness values (TRT/ORT) and the retinal sensitivity at each point were analyzed by the non-parametric Spearman test. Differences in correlation coefficient among four groups according to the retinal eccentricity were tested using the Kruskal-Wallis test. Differences in the correlation coefficient between the TRT and the ORT were tested using the Wilcoxon rank sum test. A p value of 0.05 or less was considered to indicate a statistically significant difference. The statistical analyses of the data were performed by using JMP® Pro 13.0.0 (SAS, Cary, NC).

## Results

Thirty eyes of 30 RP patients were analyzed. The characteristics, genetic information, and the examination results of the patients are summarized in Table 1. The information of each RP patient is shown in Table 2.

**Table 1:** Characteristics of the 30 patients with retinitis pigmentosa.

		median [IQR]
age, years old		41 [37–54.3]
gender, male:female		14:16
VA, Log MAR		-0.04 [-0.08–0.10]
MD with HFA, dB		-12.9 [-22.0–-4.4]
causative genes*		*EYS* (n = 3), *USH2A* (n = 1), *RHO*(n = 1), *PDE6B* (n = 1), *RP1* (n = 1), *IMPDH1* (n = 1), ND (n = 21), NT (n = 1)
retinal sensitivity with MP-3, dB		
	2°	27 [19–29]
	4°	21 [9–27]
	6°	11 [0–23]
	8°	9 [0–15]
TRT, μm, median [IQR]		
	2°	292.3 [265.9–324.1]
	4°	311.7 [274.3–340.8]
	6°	270.1 [245.2–303.3]
	8°	245.2 [220.2–273.2]
ORT, μm, median [IQR]		
	2°	58.1 [37.4–77.9]
	4°	24.9 [8.3–57.1]
	6°	12.4 [8.3–37.4]
	8°	8.3 [4.1–20.7]

*IQR*, interquartile ranges; *VA*, Visual acuity; *LogMAR*, logarithm of the minimum angle of resolution; *MD*, Mean deviation; *HFA*, Humphrey field Analyzer; *ND*, not determined; *NT*, not tested. *MP-3*, Microperimeter-3; *TRT*, total retinal thickness; *ORT*, outer retinal thickness.

*These genes were determined in the previous genomic sequence analysis of 83 RP causative genes [22].

**Table 2:** Clinical characteristics of RP patients.

	Age	Gender	Gene*	IP	VA (LogMAR)	MD value (dB)	AL (mm)	RE (diopter)
RP 1	40	F	*EYS*	AR	0.05	-26.73	24.05	-0.75
RP 2	63	M	ND	-	-0.08	-3.94	24.59	-0.25
RP 3	44	M	*EYS*	AR	0.30	-24.78	23.19	-1.4
RP 4	50	F	ND	-	0.05	-3.93	22.18	-1.25
RP 5	60	F	ND	-	-0.18	1.07	21.1	1
RP 6	37	M	ND	-	-0.08	-10.31	23.99	-1
RP 7	48	F	ND	-	-0.18	-6.97	NT	-0.4
RP 8	51	M	*USH2A*	AR	-0.08	-9.62	23.81	0
RP 9	31	M	ND	-	0.70	-25.4	25.26	-6.25
RP 10	54	F	ND	-	0.10	-15.8	NT	1.5
RP 11	26	F	ND	-	0.15	-14.91	24.06	-3.1
RP 12	25	M	NT	-	0.40	-15.83	NT	-0.9
RP 13	42	M	ND	-	-0.18	-6.02	23.73	-0.25
RP 14	38	F	ND	-	-0.08	-1.46	25.2	-3.75
RP 15	37	M	*RHO*	AD	-0.18	-14.52	NT	-1
RP 16	66	F	ND	-	0.10	-20.84	NT	3.6
RP 17	38	F	ND	-	0.00	-4.58	23.63	-2.37
RP 18	47	M	*PDE6B*	AR	0.00	-12.82	21.44	0.25
RP 19	57	M	ND	-	-0.08	-6.05	NT	0
RP 20	39	F	ND	-	0.00	-26.8	21.86	-0.5
RP 21	37	F	ND	-	0.00	-0.63	23.51	-2.62
RP 22	39	M	*RP1*	AD	0.52	-32.7	24.34	-4.8
RP 23	40	M	ND	-	-0.18	-15.1	NT	-3
RP 24	33	M	ND	-	0.00	-25.59	25.18	-1.37
RP 25	62	F	ND	-	-0.08	-3.71	24.14	-1.75
RP 26	33	F	*IMPDH1*	AD	-0.08	-12.89	NT	-1
RP 27	59	F	ND	-	-0.08	-0.5	23.11	-1.5
RP 28	44	F	ND	-	-0.08	-20.22	23.29	-2.75
RP 29	55	F	*EYS*	AR	0.10	-24.58	NT	-4.25
RP 30	33	M	ND	-	-0.08	-9.06	23.84	-1

*IP*, inheritance pattern; *VA*, visual acuity; *LogMAR*, logarithm of the minimum angle of resolution; *MD*, mean deviation with Humphry field Analyzer 10–2 program; *AL*, axial length; *RE*, refractive error; *RP*, retinitis pigmentosa; *ND*, not determined; *NT*, not tested; *AD*, autosomal dominant; *AR*, autosomal recessive.

*These genes were determined in the previous genomic sequence analysis of 83 RP causative genes [22].

Two representative cases are shown in Figs 1 and 2. The first case was a patient with the central visual acuity of 20/13. The TRT was preserved, but the ORT was thinned at the inferior 8° eccentricity. Despite the thinning of the ORT, the retinal sensitivities at the corresponding area were relatively maintained (Fig 1). The second case was a patient with the central visual acuity of 20/16 and a more advanced stage of RP compared to the above-described case. The TRT was thinned at the 8° eccentricity, and the ORT was more prominently decreased at the 4° eccentricity. The retinal sensitivity was decreased around 6° and 8° eccentricity, in line with the thinning of TRT and ORT; however, it should be noted that some retinal sensitivity remained in areas with severe ORT loss (Fig 2).

**Fig 3:**
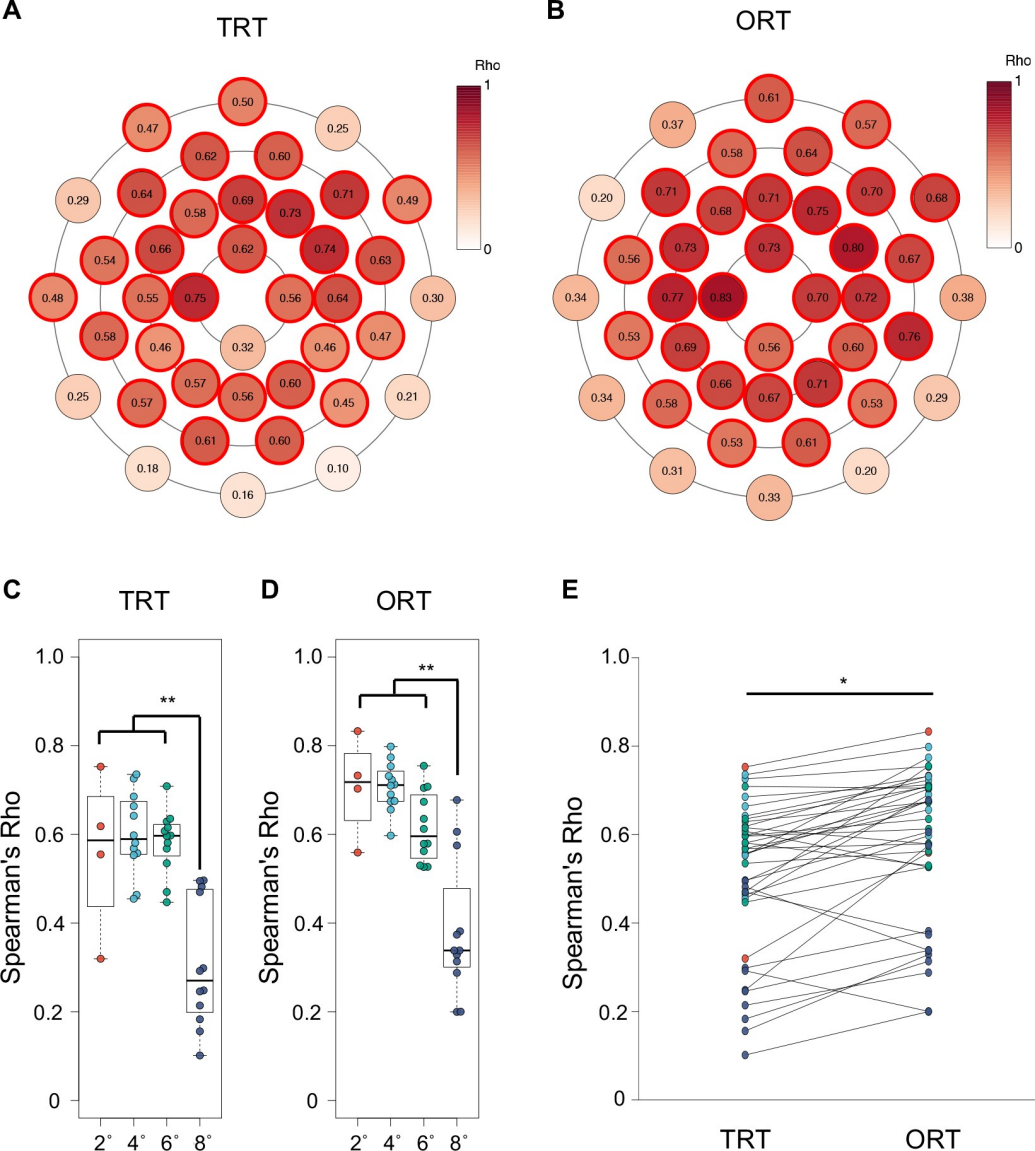
Correlation analysis between the retinal sensitivity and the TRT/ORT. **A-B:** This figure shows the rank correlation coefficients between the retinal sensitivity and the retinal thickness at each test point (**A**: TRT, **B**: ORT). The points of statistically significant correlation (p<0.05) were outlined in bright red. The intensity of the red dots indicates the strength of the correlation between the retinal sensitivity and the retinal thickness. The correlations were analyzed by the non-parametric Spearman test. **C-D:** Rank correlation coefficients of 40 points were divided into four zones according to the retinal eccentricity and the difference of correlation coefficients between 2°–6° and 8° was analyzed using the Wilcoxon rank sum test (**p<0.01) (**C**: TRT, **D**: ORT). **E:** The rank correlation coefficients between the retinal sensitivity and the ORT was compared with those between the retinal sensitivity and the TRT at each test point. The dot color indicates the distance from the fovea (orange: 2°, light blue: 4°, green: 6°, purple: 8°). The difference of correlation coefficients between two groups was analyzed using the Wilcoxon rank sum test (*p<0.05).

We next examined the correlations between the retinal sensitivity and the TRT/ORT at each retinal point (Fig 3). Both the TRT and the ORT were significantly correlated with the retinal sensitivity in all points from 2° to 6° except for one inferior point at 2° of TRT (P<0.05: Spearman test).

**Fig 4:**
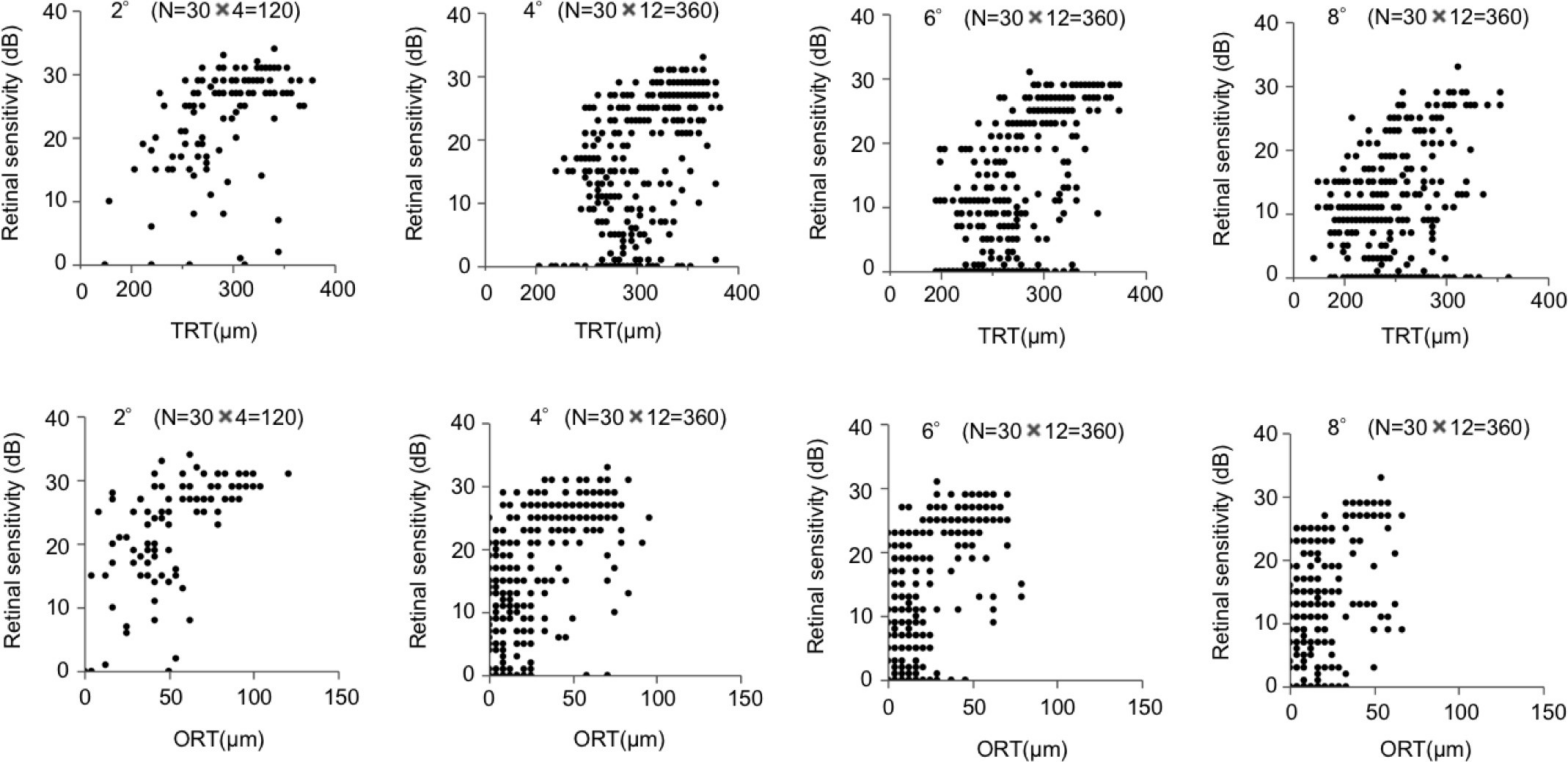
The scatter-plot analysis of the relationships between TRT/ORT and retinal sensitivity at each retinal eccentricity. The scatter-plots showing the relationships between the TRT/ORT and the corresponding the retinal sensitivity measured by MP-3 at 2°, 4°, 6°, and 8° eccentricity.

Next, we tested the difference in correlation among four groups according to the retinal eccentricity, we found significant differences in both TRT and ORT (p = 2.2×10^−4^, p = 6.3×10^−6^, respectively: Kruskal-Wallis test). The TRT showed moderate correlations with the retinal sensitivity at 2° (median ρ = 0.59 interquartile range (IQR) [0.38–0.72]), 4° (ρ = 0.59 [0.55–0.68]) and 6° (ρ = 0.60 [0.54–0.63]), and the TRT was weakly correlated with the retinal sensitivity at 8° (ρ = 0.27 [0.19–0.48]) (Fig 3). The ORT exhibited moderate-to-strong correlations at 2° (ρ = 0.72 [0.60–0.81]), 4° (ρ = 0.71 [0.67–0.75]), and 6° (ρ = 0.70 [0.54–0.74]), and a weak-to-moderate correlations at 8° (ρ = 0.34 [0.29–0.53]) (Fig 3). The correlations at 8° eccentricity appeared to be weak compared to that at 2°–6° in both the TRT and the ORT. Therefore, we analyzed the difference of the correlation coefficients between at 2°–6° and those at 8°. We found that there was a significantly strong correlation coefficients at 2°–6° compared to at those at 8° in both the TRT and the ORT (p = 1.1×10^−6^, p = 3.2×10^−6^, respectively: Wilcoxon rank sum test) (Fig 3). When compared between the TRT and the ORT, the ORT showed a significantly higher correlation coefficients with the retinal sensitivity (median ρ = 0.62 IQR [0.53–0.71]) than the TRT (ρ = 0.56 [0.45–0.62]) (p = 0.018: Wilcoxon rank sum test) (Fig 3). The scatter-plots to demonstrate the relationships between ORT/TRT and retinal sensitivity at each eccentricity are shown in Fig 4.

## Discussion

There have been several reports about the correlation between retinal thickness and visual function in various eye diseases including RP [23–26]. Witkin et al. reported that there was an excellent correlation between the distribution of the best corrected visual acuity (BCVA) and the mean foveal OS thickness in RP patients (r = −0.942, p<0.0001) [16]. Tan et al. reported that the foveal ORT (i.e., the ONL+OS thickness) was more strongly correlated with the retinal sensitivity than the foveal OS alone or the ONL alone (ORT: *r*^*2*^ = 0.36; OS: *r*^*2*^ = 0.30; ONL: *r*^*2*^ = 0.14) [27].

In the present study, we investigated the structure-function relationships in the wider regions of the macula using MP-3 and OCT, and in line with previous reports, we observed that the ORT was more strongly correlated with the retinal sensitivity compared to the TRT. These results are reasonable because photoreceptor cells are primarily injured in RP and the TRT can be affected by the thickness of inner layers, which vary depending on the distance from the fovea and can be affected by the presence of subtle epiretinal membrane and degenerative thickening [28]. Our data also showed that the structure-function correlation was stronger at 2°, 4° and 6° rather than 8° eccentricity in RP patients. In the scatter-plot analysis (Fig 4), it appears that the structure-function relationships become non-linear as the TRT/ORT decreases, suggesting that there are measurement floors for TRT/ORT. Because retinal thinning is usually more extensive in the peripheral macular region of RP patients, these floor effects may contribute to weaker correlation at 8° eccentricity. Therefore, for assessments of the macular function with multimodal methods in clinical trials of RP, consistent data may be better obtained within 6° eccentricity.

By directly comparing the retinal structure and function at the corresponding points of the retina, we expected to observe a stronger correlation than has been reported with the use of an HFA. As described in the Introduction, it is difficult to precisely overlay HFA data on a fundus image, and a study by Sayo et al. they compared the OCT structural parameters at 1°, 3°, 5°, and 7° from the fovea with the averaged retinal sensitivity at two points located above and below the target points [21]. However, contrary to our expectations, the rank correlation coefficients between the ORT and the retinal sensitivity were not much improved by our method (median ρ = 0.62 IQR [0.53–0.71]) compared with the method used by Sayo et al. (mR^2^ = 0.420) [21]. This might be explained by the lags between the appearance of the structure and functional damage as observed in glaucoma patients, in whom structural changes of the retinal nerve fiber layers precede the visual field defects [29]. Indeed, the representative cases in our study showed relatively preserved the retinal sensitivity despite the thinning of the ORT at some points (Figs 1 and 2).

There are some study limitations to consider. First, to obtain reliable MP-3 results and good-quality OCT images which are sufficient for a layer segmentation analysis, relatively younger patients with less deterioration in central vision function were included and analyzed. As a result, the structure-function relationships in advanced RP patients were not assessed in this study. Secondly, in the segmentation of the OPL layer for the ORT measurements, there were only two cases in which the OPL could be labeled by an automatic function alone, and 28 cases required manual adjustments for the analysis of RP patients. Although the ORT better reflects the visual function compared to the TRT, the process needed to create an ORT map is still time-consuming and currently not usable in busy clinical settings. Thirdly, since the examinations were performed on only a single occasion for each patient, the longitudinal correlations between the changes in retinal thickness and sensitivity are currently unknown. Long-term observations of the structure-function relationships may provide more valuable information for the disease evaluation in clinical settings and trials.

In conclusion, the ORT, rather than the TRT, within 6° eccentricity was strongly correlated with the retinal sensitivity, suggesting that measuring the ORT in those areas would be useful for evaluating the macular status and progression in patients with RP. However, despite our direct structure-function comparison, the parameters did not exactly correlate, suggesting that there are certain lags between the retinal thinning and sensitivity loss.
